# Skip DETR: end-to-end Skip connection model for small object detection in forestry pest dataset

**DOI:** 10.3389/fpls.2023.1219474

**Published:** 2023-08-15

**Authors:** Bing Liu, Yixin Jia, Luyang Liu, Yuanyuan Dang, Shinan Song

**Affiliations:** ^1^ College of Computer Science and Technology, Jilin University, Changchun, Jilin, China; ^2^ School of Computer Science and Engineering, Changchun University of Technology, Changchun, Jilin, China

**Keywords:** object detection, forestry pest detection, DETR, Skip connection, small object detection

## Abstract

Object detection has a wide range of applications in forestry pest control. However, forest pest detection faces the challenges of a lack of datasets and low accuracy of small target detection. DETR is an end-to-end object detection model based on the transformer, which has the advantages of simple structure and easy migration. However, the object query initialization of DETR is random, and random initialization will cause the model convergence to be slow and unstable. At the same time, the correlation between different network layers is not strong, resulting in DETR is not very ideal in small object training, optimization, and performance. In order to alleviate these problems, we propose Skip DETR, which improves the feature fusion between different network layers through skip connection and the introduction of spatial pyramid pooling layers so as to improve the detection results of small objects. We performed experiments on Forestry Pest Datasets, and the experimental results showed significant AP improvements in our method. When the value of IoU is 0.5, our method is 7.7% higher than the baseline and 6.1% higher than the detection result of small objects. Experimental results show that the application of skip connection and spatial pyramid pooling layer in the detection framework can effectively improve the effect of small-sample obiect detection.

## 1 Introduction

Object detection is one of the more important branches in the field of computer vision Zaidi et al. (2022), and it has been widely used in agricultural pest detection, crop condition detection, crop yield prediction, and other fields. In recent years, with the vigorous development of deep convolutional neural networks, the accuracy and performance of object detection tasks have been greatly improved. The identification and detection of forest pests provide a strong guarantee for crop yield growth and the agricultural economy Ngugi et al. (2021). However, compared with tasks such as autonomous driving and intelligent monitoring, forestry pest detection still has the following challenges: (1) There are fewer publicly available datasets for forest pests; (2) The detection targets are mostly small targets Huang et al. (2022).

At present, there are few studies on forest pest detection, and forestry pest datasets are relatively lacking. Most of the proposed solutions rely on traditional CNN structures, such as ResNet, GoogleNet, VGG, etc. The root cause of this problem is the lack of a large enough dataset of forestry pests to train specific CNN structures Ngugi et al. (2021). These traditional architectures have high computational requirements for pest identification tasks and require high-resolution image features in processing small target images, which will bring high computational complexity. Therefore, Carion et al. (2020) proposed an end-to-end object detection model (DETR) based on Transformers and achieved competitive results. The main hurdle in forestry pest datasets is that the only large, freely available datasets are the PlantVillage dataset and the Plant Disease Symptom Image Database (PDDB). The recently published forestry pest dataset solves the above problems well. The dataset contains 7163 images and 31 species of forestry pests Liu et al. (2022a).

Current mainstream non-end-to-end object detection frameworks include one-stage and two-stage detectors. They may face a huge amount of computation, which can greatly improve model training time and performance Sun et al. (2021a). The DETR architecture Carion et al. (2020), as an end-to-end object detection framework, has a simple structure and does not require specialized libraries. This means faster setup of deployments or downstream tasks on other computers. At the same time, experiments show that DETR also has high performance when migrating to other tasks, such as panoramic segmentation.

However, DETR, a method that uses object queries matching, usually requires high costs when performing intensive detection. There are many ways to improve DETR, such as the way Deformable DETR uses Deformable’s structure and Multi-Scale Zhu et al. (2020). It greatly reduces the training cost of the model while improving the performance of the model. At the same time, due to the lack of image priori and multi-scale fusion mechanisms of DETR Liu et al. (2022b), although recent DETR-based models have achieved significant performance, DETR lacks multi-scale features compared to classical object detection models, which are critical for small object detection.

As a classical method to improve the structure of deep neural networks, skip connection He et al. (2016) has been applied in classical network structures such as U-Net, ResNet, and DenseNet, which plays a role in improving the accuracy of image segmentation and improving the utilization rate of feature information in each layer of the network. Therefore, this paper introduces skip connection and spatial pyramid pooling layers He et al. (2015) to enhance the extraction and fusion of image features by the model and enhance the model’s learning of small object objects. First, we link the backbone network output and encoder output with skip connection. Then, in the DETR decoder input, 100 randomly initialized object queries are included, but random initialization also makes image feature learning slow Chen et al. (2022). So we use a three-layer spatial pyramid pooling layer to transform the output of the backbone network into features of the same size as the object queries, and finally perform skip connection to improve the initialization process of the object queries.

With our improved method, we have improved the accuracy of small target detection. At the same time, compared with the initial DETR model, our model has achieved competitive results. On Forestry Pest Datasets, at IoU=0.5 and IoU=0.75, our method achieves an absolute gain of 7.7% AP and 6.0% AP on the DETR baseline. For small object detection results, our method achieves a gain of 6.1% AP over the DETR baseline.

The contribution of this work are summarized as follows:

1) We propose a model called Skip DETR, which uses skip connection to enhance the extraction of features of small sample images by the DETR model.
2) We introduce the spatial pyramid pooling layer, improve the object queries initialization method, and make the model converge faster.
3) We conduct extensive experiments on forestry pest datasets. Experimental results show that the application of skip connection and spatial pyramid pooling layer in the detection framework can effectively improve the effect of small-sample object detection.

## 2 Related works

In this section, we will introduce the relevant solutions for insufficient multi-scale feature fusion and the research status of small target detection, identify and review existing forest pest datasets and related detection methods.

### 2.1 A solution to insufficient multi-scale feature fusion

The DETR architecture, as an end-to-end object detection framework, has a simple structure and does not require specialized libraries. However, the DETR model does not contain FPN, resulting in high computational complexity and insufficient feature fusion when processing high-resolution image features. However, DETR requires high-resolution image features when processing small target images, which brings high computational complexity. Therefore, it is not suitable to introduce FPN inside the DETR model, which ultimately leads to insufficient feature fusion. Without reintroducing multiscale feature fusion in the encoder, the accuracy of DETR cannot be further improved. Therefore, six Transformer encoder layers are included in the DETR encoder, which are stacked on top of the backbone network to improve the feature representation of its model.

At present, many improved models of DETR are trying to solve this problem. Deformable DETR combines DCN sparse sampling capabilities with transformer global relationship modeling capabilities by using the Deformable Attention module Zhu et al. (2020). Sun et al. (2021b) solved the cross-attention problem in DETR by proposing two schemes, TSP-FCOS and TSP-RCNN. The ViT-FRCNN model improves the structure of DETR by replacing the transformer with the backbone portion of FasterRCNN Beal et al. (2020). Since the introduction of FPN is not suitable inside DETR, the work in this paper is mainly to add skip connections and SPP networks outside DETR to enhance the fusion of its multi-scale features.

### 2.2 Public datasets of forest pests and pest object detection

The identification and detection of pests and diseases provide a strong guarantee for crop yield growth and agricultural economy in forestry pest control. Current forestry pest datasets can provide a wide variety of training samples for target detectors. Sun et al. (2018), as well as Hong et al. (2021), used pheromone traps to collect datasets that created forestry pests, but the datasets they created were only able to handle specific species of forestry pests. Chen et al. (2019) also created a dataset of forestry pests, but their main purpose was to study the classification of pests. Baidu has also published a dataset of forestry pests, but it was collected in a lab-built environment. Therefore, finding a public and suitable forestry pest dataset is difficult. However, the forestry pest dataset recently published by Liu et al. (2022a), which contains 31 pests and more than 7,000 images, lays a good foundation for the training of target detectors in this field.

Early pest and disease object detection was largely based on machine learning techniques. Le-Qing and Zhen (2012) tested 10 pests on a dataset of 579 samples using local average color features and SVMs.

Zhang et al. (2013) proposed a field pest identification system, and the dataset they used included about 270 training samples. Ebrahimi et al. (2017) used an SVM method with differential kernel functions for parasite classification and thrips detection. These early pest detection methods have yielded good results. However, their detection performance depends on the performance of the manual feature extractor and the chosen classifier.

With the development of image technology, convolutional neural networks have achieved obvious advantages in complex object detection, segmentation and classification by virtue of their strong image feature learning ability. Selvaraj et al. (2019) constructed an AI-based banana pest detection system based on deep convolutional neural network (DCNN). Liu and Wang (2020) constructed a tomato pest dataset and improved the YOLOV3 model to detect tomato pests and diseases based on this dataset. Zhu et al. (2021) improved the YOLOV3 model for the detection of black rot in grape leaves using super-resolution image enhancement.

In summary, although convolutional neural network (CNN)-based pest detection can improve the performance of pest detection, it has the advantage of avoiding the early limitations of the model. However, the fly in the ointment is that the vast majority of object detection architectures have manually designed components that have an impact on the performance of the model. Recently, the end-to-end object detection model (DETR) based on Transformers proposed by Carion et al. (2020) can avoid the above problems well and achieve competitive results.

### 2.3 Status of small target detection

Small target detection plays an important role in forestry pest detection, crop status detection, crop yield prediction and other scenarios Du et al. (2022). Small target detection has the characteristics of small coverage image area, few effective features for object detection, and commonly used object detectors are insensitive to small targets. At present, for small target detection, MR-CNN adopts multi-scale feature fusion Gidaris and Komodakis (2015), ContextNet uses context information to improve R-CNN Han et al. (2020), JCS-Net adopts image super-resolution and other methods Pang et al. (2019), which has been improved in the detection results of small targets.

However, most studies did not work on forest pest datasets, and there are still some gaps in the detection of small targets in the field of forest pest identification.

## 3 Framework of Skip DETR model

In this section, we will introduce the model structure of Skip DETR and elaborate on the structure of the improved components and how they provide gain to the model.

### 3.1 The structure of Skip DETR

Skip DETR is an improved end-to-end object detection framework based on DETR, which mainly includes three parts: backbone, transformer-based encoder-decoder structure, and sequence prediction architecture. At the same time, we add a spatial pyramid pooling layer and a deep separable convolutional layer outside the DETR model, and enhance the fusion of contextual feature information through skip connections.

**Figure 1:**
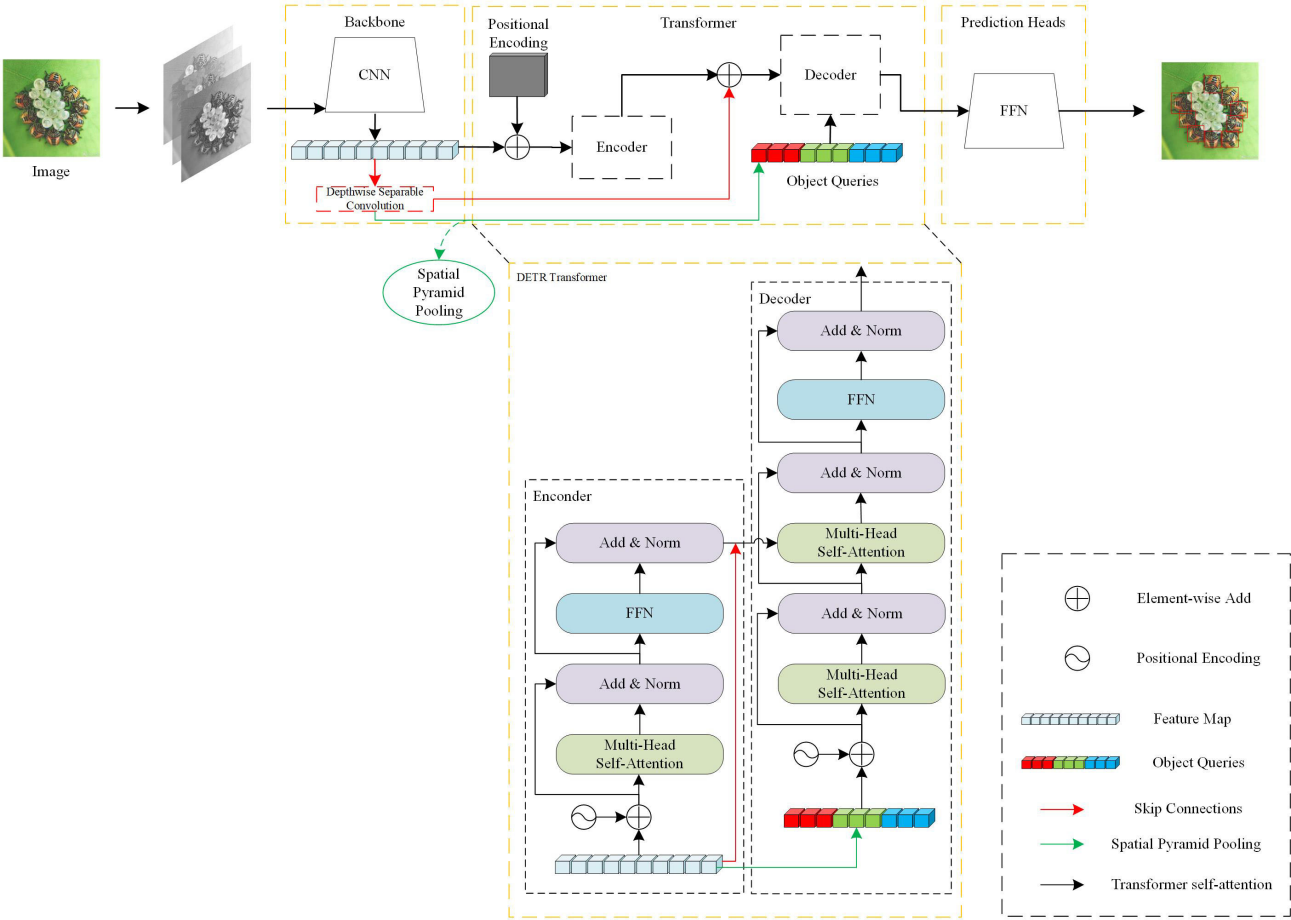
The structure of Skip DETR. Our work is mainly carried out outside the DETR model. We introduce a deep separable convolutional layer and a spatial pyramid pooling layer, and skip connection with the encoder output and object queries.

When the image is input to the model, it will first be processed by the CNN to obtain the feature matrix of the current image. Then the feature matrix will be straightened and added to the position encoding, and passed into the encoder to learn the global information of the image, and the straightened feature matrix will be further extracted by deep separable convolutional layers. The results of the subsequent deep separable convolutional layer processing will be residually connected to the Encoder output on the one hand, and a three-layer spatial pyramid pooling layer and connected to the object query as the input of the Decoder on the other hand. Finally, it is decoded by Decoder and passed to FFN for image prediction. We will show the structure of our Skip DETR in **Figure 1**.

### 3.2 Applying skip connections between different layers of DETR

The basic idea of skip connection is to express the output as a linear superposition of a nonlinear transformation of the input and output He et al. (2016). After the skip connection, the amount of information describing the characteristics of the image increases, but the feature dimension describing the image does not change. Because the amount of information increases in each dimension, it is beneficial for the final image prediction.

When we use a non-linear change function to describe the input and output of a network, that is, the input is x and the output is F(x), F usually includes operations such as convolution and activation. When we add an input to the output of a function, although we can still use G(x) to describe the relationship between input and output, G(x) can be explicitly split into linear overlays of F(x) and X.

Srivastava et al. (2015) proposed the residual structure for the first time, which is derived from the control gate idea of LSTM. The initial residual formula is shown in formula 1.


(1) $$\text{y}=\text{H}(\text{x},{\text{WH}}_{\;})\cdot \text{T}(\text{x},{\text{WT}}_{\;})+\text{x}\cdot (1-\text{T}(\text{x},{\text{WT}}_{\;}))$$

However, because Formula 1 is too complex, He et al. (2016) simplified the formula, which is shown in formula 2.


(2) $$\text{y}=\text{H}(\text{x},{\text{WH}}_{\;})+\text{X}$$

**Figure 2:**
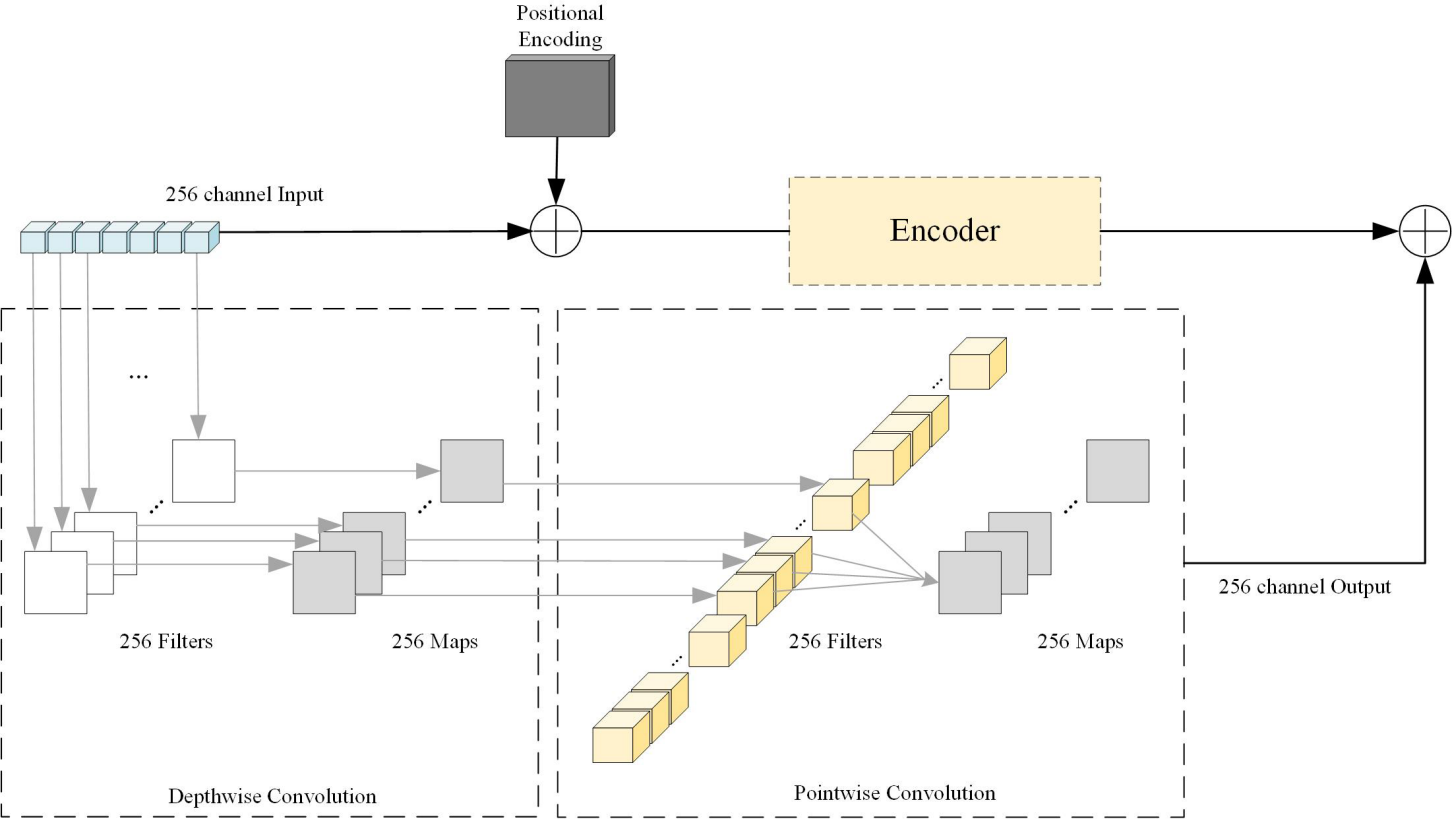
Depthwise Separable Convolution and residual structure diagram. Depthwise Separable Convolution is divided into two parts: Depthwise Convolution and Pointwise Convolution. We set the relevant parameters according to the input image features. It is worth noting that the convolution kernel size of both parts is 1×1.

Inspired by the residual structure, this paper applies the residual structure to the DETR network. On the one hand, we link the backbone network output with the encoder output to enhance the learning of small objects by fusing image features. In order to reduce the number of model parameters and operation costs,

we introduce a depthwise separable convolutional layer after the output of the backbone network. We set the convolution kernel size to 1, the stride to 1, the depthwise part group to 256, and the pointwise part group to 1. We will show the structure of this part of the component in **Figure 2**.

On the other hand, the decoder input of DETR contains 100 randomly initialized object queries, but random initialization also makes the model converge slowly. So we want to link the backbone network output with the object query to speed up the convergence of the model by improving the initialization of the object query. However, since the backbone network output feature map size is variable, it is not possible to unify the output feature map size using a common convolution layer. This paper solves this problem by introducing a spatial pyramid pooling layer, which will be described in the next section.

### 3.3 Add a spatial pyramid pooling layer

**Figure 3:**
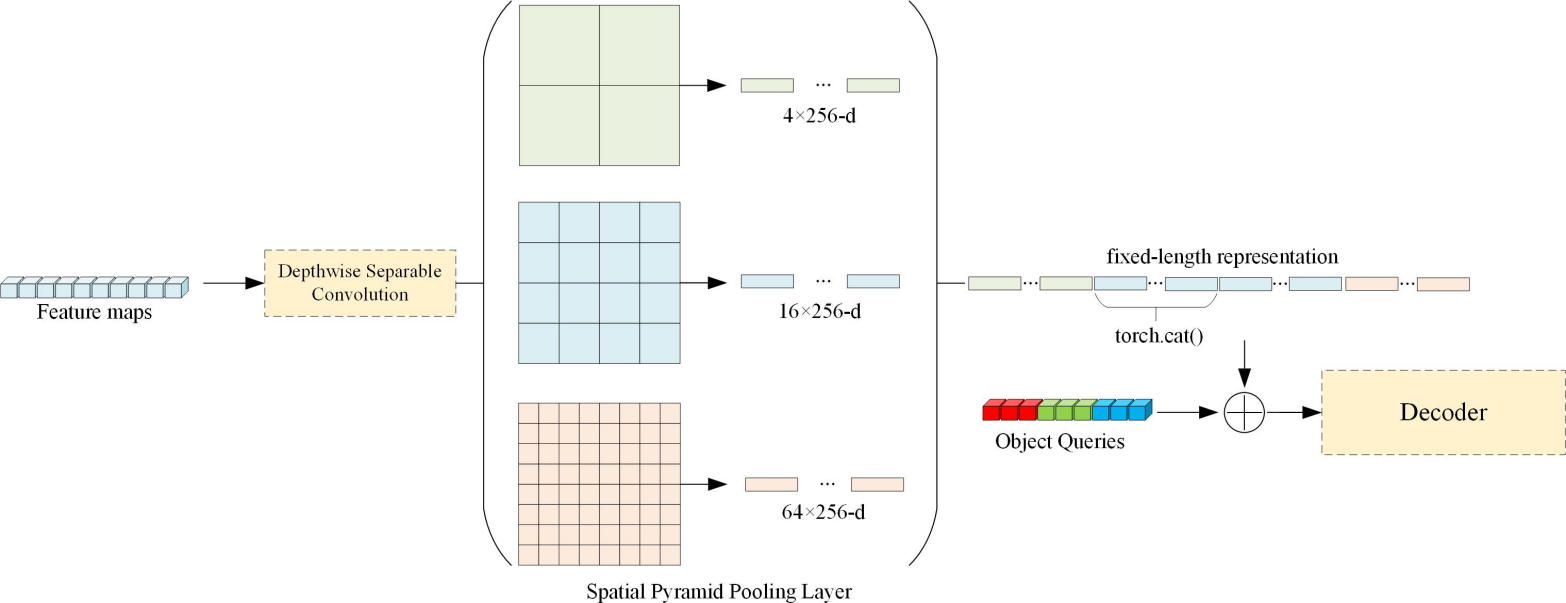
Spatial pyramid pooling layer structure diagram. We design a three-layer spatial pyramid pooling layer, decompose the feature map into 2×2, 4×4, 8×8 sizes, and at the same time stitch the output of the second layer twice when unfolding and stitching. Finally, we connect the output with the object queries.


The essence of the pooling layer of the spatial pyramid is the multi-layer maximum pooling layer, which generates a fixed-size output for feature maps (*n* × *n*) of different sizes (*α* × *α*). The spatial pyramid pooling layer automatically adjusts the size of the sliding window win and the step size str according to different input sizes, using Equation 3 and Equation 4. In this paper, the output results of deeply separable convolutional layers are processed by spatial pyramid pooling layers and residually connected with object queries in the DETR model. This component changes the initial state of the object queries, providing the model with *a priori* information that can learn the key features of the image, thereby shortening the convergence process of the model. We will show the structure of this component in **Figure 3**.

(3) win=ceil(α/n)

(4) str=floor(α/n)

Many CNN models have certain requirements on the size of the input image Long et al. (2015), but the feature extraction network (such as convolution layer, activation function layer, pooling layer) part of the model head has no requirements on the input image, which can be simply understood as the feature extraction network knowledge reduces the image by a fixed multiple. However, the full connection layer at the end of the model has strict requirements on the input dimension. Therefore, limiting the image size of the input CNN model is to meet the requirements of the full connection layer.

In the decoder input of the DETR, 100 randomly initialized object queries are included, and the size of each object query is 100 × 256. However, the size of the feature map output by the backbone network cannot be guaranteed to be the same as the size of object query. Therefore, we first pass the output of the backbone network through a Depthwise Separable Convolutional layer (the convolutional layer is described in Section 3.1), and then pass through a three-layer spatial pyramid pooling layer, so that the final output can meet the skip connection condition with the object query.

In the process of using the pooling layer of the spatial pyramid, we also hope to integrate the outputs of different pooling layers. Therefore, we integrate the output of the second pooling layer with the output of the third pooling layer, hoping to further enhance the model’s ability to extract features.

## 4 Experiment

We show that in the quantitative assessment of Forestry Pest Datasets, our improved DETR has achieved competitive results compared with the baseline of the DETR. Then we carried out a detailed ablation study and gave qualitative results.

### 4.1 Parameters of model training

The experiments used in this paper is Python 3.9, Torch 1.11, CUDA 11.4. Other hardware information used in the experiment is shown in **Table 1**. The main parameters of the Skip DETR model are shown in **Table 2**. DETR requires about 500 epochs on the COCO dataset to converge, considering the detection accuracy and training time on the Forestry Pest dataset, we choose 300 epochs. At the same time, in order to ensure the consistency of the training cycle of the control experiment, we designed the same epoch for other experiments. If there is no special declaration, all other parameters are consistent with the DETR.

**Table 1:** Configuration of experimental environment.

Hardware	Model
CPU	Silver 4110
Memory	64GB
GPU	Quadro P2000 5GB
Hard disk	2.5TB

**Table 2:** Model parameter settings of Skip DETR.

Name	Value
Batch size	1
Epoch	300
Learn rate	0.00001

### 4.2 Dataset

At present, there are many datasets related to the Forestry Pest identification task (such as Hong et al. (2021); Chen et al. (2019); Sun et al. (2018)), but they have problems such as few pest species, being unable to apply to the actual scene, and the data set is not open to the public. However, the recently published Forestry Pest dataset has solved the above problems well Liu et al. (2022a). The dataset contains 7163 images and 31 forest pests. The dataset is derived from Liu et al. (2022a), and the types and quantities of forest pests in the dataset are shown in **Table 3**.

**Table 3:** Details of the types and quantities of forest pests in the dataset.

Class index	Pest	Sample size
0	Drosicha contrahens (female)	218
1	Drosicha contrahens (male)	210
2	Chalcophora japonica	158
3	Anoplophora chinensis	426
4	Psacothea hilaris(Pascoe)	218
5	Apriona germari(Hope)	342
6	Monochamus alternatus	184
7	Plagiodera versicolora(Laicharting)	306
8	Latoia consocia(Walker)	290
9	Hyphantria cunea	303
10	Cnidocampa flavescens(Walker)	290
11	Cnidocampa flavescens(Walker) (pupa)	176
12	Erthesina full	280
13	Erthesina fullo (nymph)	156
14	Erthesina fullo (nymph2)	192
15	Spilarctia subcarnea(Walker)	188
16	Psilogramma menephron	218
17	Sericinus montela	364
18	Sericinus montela (larvae)	200
19	Clostera anachoreta	294
20	Micromelalopha troglodyta(Graeser)	238
21	Latoia consocia(Walker) (larvae)	204
22	Plagiodera versicolora(Laicharting) (larvae)	196
23	Plagiodera versicolora(Laicharting) (ovum)	134
24	Spilarctia subcarnea(Walker) (larvae)	186
25	Spilarctia subcarnea(Walker) (larvae 2)	164
26	Psilogramma menephron (larvae)	208
27	Cerambycidae (larvae)	196
28	Micromelalopha troglodyta(Graeser) (larvae)	226
29	Hyphantria cunea (larvae)	224
30	Hyphantria cunea (pupa)	174

Therefore, we use the dataset of Liu et al. (2022a) for training. In order to ensure the training results, the Forestry Pest dataset is randomly divided according to the following proportion: (Train: Val=9:1): Test=9:1. That is, 5801 training images, 645 verification images and 717 test images for target detection tasks are included after division.

### 4.3 Evaluation metrics

In this paper, we use mAP and AR as experimental evaluation indicators, which are widely used in the field of object detection. We will give the calculation method of mAP and AR.


(5) $$\text{Precision}=\frac{TP}{TP+FP}$$

(6) $$\text{Recall}=\frac{TP}{TP+FN}$$

(7) $$\text{mA}{\text{P}}_{\alpha }=\frac{1}{N}\sum_{n=1}^{N}AP_{\alpha }^{n}$$

where *TP* for positive samples is predicted as positive class, *FP* is negative samples are predicted as positive class, and *FN* is positive samples are predicted as negative class. *AP* is the average accuracy, which is simply the average of the Precision value on the *PR* curve, and *mAP_α_* represents the *AP* measurement at different IoU thresholds.

In the COCO dataset, objects with a pixel area less than 32 *×* 32 are regarded as small objects, pixel faces and objects larger than 96 *×* 96 are regarded as large objects, and pixel faces and objects between 32 *×* 32 and 96 *×* 96 are regarded as medium objects.

### 4.4 Experimental results

Skip DETR is an improvement based on the DETR model and is mainly designed for small target detection.

We try to improve the performance improvement of DETR on small target detection with our method. The average accuracy of the Skip DETR model at different IoU Rezatofighi et al. (2019) thresholds, the results are shown in **Table 4**.

**Table 4:** The average accuracy of the Skip DETR model at different IoU thresholds.

Model	GFLOPS	#params	Epoch	*AP*	*AP*_50_	*AP*_75_
DETR	5.13	36.7M	100	52.3	67.7	59.1
Ours	5.14	36.8M	100	56.1	74.4	62.4
DETR	5.13	36.7M	200	57.5	73.3	62.4
Ours	5.14	36.8M	200	65.8	82.1	70.8
DETR	5.13	36.7M	300	65.7	79.1	71.0
Ours	5.14	36.8M	300	**74.1**	**86.8**	**77.0**

From the experimental results in **Table 4**, it can be seen that after training with 300 epochs, the Skip DETR model has better accuracy than the DETR model on the forest pest dataset. When IoU=0.5, The 200th epoch result of Skip DETR is even higher than the 300th epoch result of the DETR model. At the same time, the results on other evaluation indicators are also due to the DETR model. This shows that the improvement method we use helps to improve the accuracy of the model.

Another drawback of DETR is its poor performance in detecting small objects. To verify whether our model helps improve the accuracy of small object detection, we compare the detection accuracy of Skip DETR and DETR at different scales. The results are shown in **Table 5**.

**Table 5:** The detection accuracy of Skip DETR and DETR at different scales.

Model	Epoch	*AP_S_*	*AP_M_*	*AP_L_*
DETR	100	0.7	8.8	59.4
Ours	100	1.0	12.3	63.1
DETR	200	1.2	12.8	64.3
Ours	200	7.6	22.5	73.3
DETR	300	6.0	21.1	73.1
Ours	300	**12.1**	**32.4**	**81.9**

As can be seen from the results in **Table 4**, our model is a significant improvement in the detection of small objects. After 300 epochs, compared with DETR, the accuracy of skip DETR in small object detection is improved by 6.1% AP, the medium object detection accuracy is improved by 11.3% AP, and the detection accuracy of large objects is improved by 8.8% AP.

Recall is often used to assess detector coverage of all objects to be inspected Buckland and Gey (1994).Therefore, we compared the recall of Skip DETR and DETR at different training stages and scales in **Table 6**. We selected 100 subjects to test the average recall, and the final result showed that Skip DETR can predict positive samples more accurately.

**Table 6:** The recall of Skip DETR and DETR at different training stages and scales.

Model	Epoch	MaxDets	*AR_S_*	*AR_M_*	*AR_L_*
DETR	100	100	7.2	23.1	74.0
Ours	100	100	8.2	30.0	77.8
DETR	200	100	13.0	35.0	77.6
Ours	200	100	18.4	4.06	82.3
DETR	300	100	21.3	39.0	81.1
Ours	300	100	**27.1**	**51.4**	**87.6**

Our improved DETR model has obtained good results, especially greatly improved AP*_S_*, AP*_M_* and AP*_L_*.

Finally, we used the original DETR model and the improved DETR model to measure GFLOPS Goldberg (1991) and params. Through experiments we find that the improvement method proposed in this paper is completely negligible in terms of consumption of computing resources.

**Figure 4:**
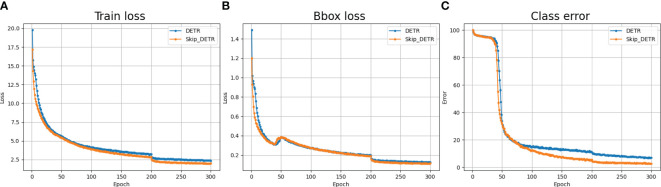
Comparison of common model evaluation indicators. **(A)** *Training loss comparison*; **(B)** *Box loss comparison*; **(C)** *Class error comparison*.

During model training, training loss Ru et al. (2020), box loss, and classification error rate Kim (2009) are common metrics to measure model performance. In **Figure 4** we show the comparison results of the Skip DETR and DETR models on the above evaluation indicators. From **Figure 4**, it can be seen that the loss error of Skip DETR in the initial training phase is lower than that of the DETR model. As training progresses, Skip DETR’s loss and classification error rate decreases faster than DETR. This indicates the effectiveness of adding skip connection and spatial pyramid pooling layers. In addition, the training process of Skip DETR is smoother, which is easier to train than the DETR model.

Furthermore, in **Table 7**, we compare our Skip DETR with several different object detection models on Forestry Pest Datasets. We report the detection results of each model at different IoU thresholds and scales. For fair comparison, we used the same model parameters and trained the same epochs. We show that Skip DETR outperforms DETR and achieves competitive results compared with other object detection models and improved models based on DETR.

**Table 7:** Ablation study for each part of the contribution to DETR.

Model	*AP*	*AP*_50_	*AP*_75_	*AP_S_*	*AP_M_*	*AP_L_*
DETR	57.5	73.3	62.4	1.2	12.8	64.3
UP-DETR	59.1	76.3	63.8	6.3	18.5	65.4
YOLOV3	51.0	81.8	56.5	7.2	22.3	54.8
Skip DETR (ours)	65.8	82.1	70.8	7.6	22.5	73.3

### 4.5 Ablation experiments

In this section, we conducted several ablation experiments to help us understand the contribution of each improved method to the final performance. As shown in **Table 8**, both improvement methods improve the performance of DETR.

**Table 8:** Ablation study for each part of the contribution to DETR.

Model	#params	Epoch	*AP*	*AP*_50_	*AP*_75_	*AP_S_*	*AP_M_*	*AP_L_*
DETR	36.7M	300	65.7	79.1	71.0	6.0	21.1	73.1
DETR+SPP	36.8M	300	63.4	74.8	65.8	8.3	19.9	72.7
DETR+skip connection+SPP(ours)	36.8M	300	74.1	86.8	77.0	12.1	32.4	81.9

It is worth noting that we first introduced the spatial pyramid pooling layer on the basis of DETR, and we found that although it provides performance improvement for DETR in the recognition of small objects, it will reduce the performance of other indicators. Therefore, we introduced the skip connection on the basis of the first improvement, and experimented with the skip connection and the spatial pyramid pooling layer as a whole module, and finally obtained the performance improvement on all indicators while the number of model parameters remained basically unchanged.

In general, in the field of forestry pest detection and small target detection, Skip DETR adds skip connections and spatial pyramid pooling layer so that our model can make full use of the image information in the feature map at various scales, and making the model more sensitive to small targets. At the same time, the spatial pyramid pooling layer can change the initialization mode of object queries, making the convergence of Skip DETR models faster and easier to train. Without changing the number of model parameters too much, Skip DETR has achieved competitive results on multiple evaluation indicators. And compared to several other different object detection models, Skip DETR also achieves better results.

## 5 Conclusion

In this work, we propose a model called Skip DETR, which uses skip connection to enhance the extraction of image features from small samples by the DETR model. At the same time, we introduce the spatial pyramid pooling layer, improve the object query initialization method, and make the model converge faster. Finally, we conduct extensive experiments on forestry pest datasets. Experimental results show that the application of skip connection and spatial pyramid pooling layer in the detection framework can effectively improve the effect of small-sample object detection.

Although Skip DETR achieved good results, our study still faced the problem of small data pools. At the same time, in order to improve the detection accuracy, we will continue to improve the Skip DETR model.

## Data availability statement

The original contributions presented in the study are included in the article/supplementary files, further inquiries can be directed to the corresponding author.

## Author contributions

BL and YJ conducted experiments, analyzed data, and wrote manuscripts. LL provided a dataset of forestry pests. YD and SS designed the research and revised the manuscript. All authors contributed to the article and approved the submitted version.
